# Prevalence of chronic kidney disease and comorbidities in isolated African descent communities (PREVRENAL): methodological design of a cohort study

**DOI:** 10.1186/s12882-018-0839-z

**Published:** 2018-02-26

**Authors:** Natalino Salgado-Filho, Joyce Santos Lages, Dyego José Brito, João Victor Salgado, Gyl Eanes Silva, Alcione Miranda Santos, Francisco Chagas Monteiro-Júnior, Elisangela Milhomen Santos, Antônio Augusto Silva, Denizar Vianna Araújo, Ricardo Castro Sesso

**Affiliations:** 1 Kidney Disease Prevention Centre and Department of Medicine I, Federal University of Maranhão, São Luís, MA Brasil; 2 Department of Public Health, Federal University of Maranhão, São Luís, MA Brazil; 3 Kidney Disease Prevention Centre and Department of Physiological Sciences, Federal University of Maranhão, São Luís, MA Brazil; 4 Department of Pathology and Radiology, Ribeirao Preto School of Medicine, University of Sao Paulo, Ribeirão Preto, SP Brazil; 5 Department of Internal Medicine, Rio de Janeiro State University, Rio de Janeiro, RJ Brazil; 6 Discipline of Nephrology, Paulista School of Medicine, Federal University of São Paulo, São Paulo, SP Brazil

**Keywords:** African, Kidney disease, Glomerular filtration rate, Brazil, Population study

## Abstract

**Background:**

Chronic kidney disease (CKD) is considered a serious public health problem, both in Brazil and worldwide, with an increasing number of cases observed inrecent years. Especially, CKD has been reported to be highly prevalent in those of African descent. However, Brazil lacks data from early-stage CKD population studies, and the prevalence of CKD is unknown for both the overall and African descent populations. Hence, the present study aimsto estimate the prevalence of early-stage CKD and its associated risk factors in African-Brazilians from isolated African-descent communities. Herein, the detailed methodology design of the study is described.

**Methods:**

This population-based, prospective, longitudinal, cohort study (PREVRENAL) is performed in three stages: first, clinical, nutritional, and anthropometric evaluations; measurements of serum and urinary markers; and examinations of comorbiditieswere performed. Second, repeated examinations of individuals with CKD, systemic arterial hypertension, and/or diabetes mellitus; image screening; and cardiac risk assessment were performed. Third, long-term monitoring of all selected individuals will be conducted (ongoing). Using probability sampling, 1539 individuals from 32 communities were selected. CKD was defined asaglomerular filtration rate (GFR) ≤60 mL/min/1.73m^2^ and albuminuria > 30 mg/day.

**Discussion:**

This study proposes to identify and monitor individuals with and without reduced GFR and high albuminuria in isolated populations of African descendants in Brazil. As there are currently no specific recommendations for detecting CKD in African descendants, four equations for estimating the GFR based on serum creatinine and cystatin C were used and will be retrospectively compared. The present report describes the characteristics of the target population, selection of individuals, and detection of a population at risk, along with the imaging, clinical, and laboratory methodologies used. The first and second stages have been concluded and the results will be published in the near future. The subsequent (third) stage is the long-term, continuous monitoring of individuals diagnosed with renal abnormalities or with CKD risk factors. The entire study population will be re-evaluated five years after the study initiation. The expectation is to obtain information about CKD evolution among this population, including the progression rate, complication development, and cardiovascular events.

## Background

### The clinical problem

Chronic kidney disease (CKD) is considered a serious public health problem in Brazil and worldwide, with an increasing number of cases during recent years [1, 2]. Brazil still lacks data from population studies concerning early stage CKD. The available information is restricted to those from patients with stage 5 CKD, who received renal replacement therapy in dialysis units [3, 4]. Approximately 40% of the Brazilian population is of African descent, which ranks Brazil as the county with the second highest percentage of African descendants worldwide (following Nigeria). From a social-economic perspective, African descendants are poorer and less educated when compared with the overall population. Studies of this population group in other countries, primarily the United States, suggest that kidney diseases are more prevalent in those of African descent, especially CKD, when compared with other ethnic groups [5–8]. Ethnic studies also demonstrated high rates of adverse cardiovascular events [9, 10], diabetes mellitus (DM), and obesity, among other risk factors [11, 12], among this group. It is worth emphasizing that even during the early stages of CKD,with discrete decreases in glomerular filtration rate (GFR), cardiovascular diseases (CVD) are the primary causes of death [13].

### Aims of this study

The present study (PREVRENAL) aimedto estimate the prevalence of earlystage CKD and its associated risk factors in African Brazilians from isolated quilombo communities. It also aims to evaluate demographic, clinical, and laboratory aspects of this patient population, and identify factors associated with the development of CVD.

## Methods and study design

### Study location and population

This study was performed in Alcântara, Brazil, a city located on the west coast of the State of Maranhão (Northeastern Brazilian region). This city has 21,851 inhabitants, with 15,452 individuals (70.71%) from rural populations distributed among 139 isolated African-descent communities (quilombos). Ancestors of the evaluated ethnic group were from the Ivory Coast, Angola, and Mozambique. Criteria used for ethnicity definition was the same as that used by the Brazilian Geography and Statistics Institute, which adopts self-assignment, posteriorly confirmed by genetic examination.

The sample individuals of this study were selected through probability sampling, which was designed in three stages. First, there was a randomized selection of census sectors, with probabilities proportional to population sizes. Second, households were randomly selected. Third, individuals aged over 18 years old, who lived in the selected households, were included in the study sample. Total sample size was estimated per 15% CKD prevalence expectation, with 2% sampling error, and 95% confidence level, composing a minimal sample of 1224 individuals. Finally, 1539 individuals were selected from 32 quilombo communities. The following were excluded from the sample: individuals under 18 years old, pregnant women, and patients with chronic consumptive disease, hematological diseases, autoimmune diseases, infections, chronic or acute kidney diseases undergoing dialysis treatment, thyroid disease, and those using immunosuppressant drugs, based on clinical history and physical examination.

### Study design and summary

**Fig. 1:**
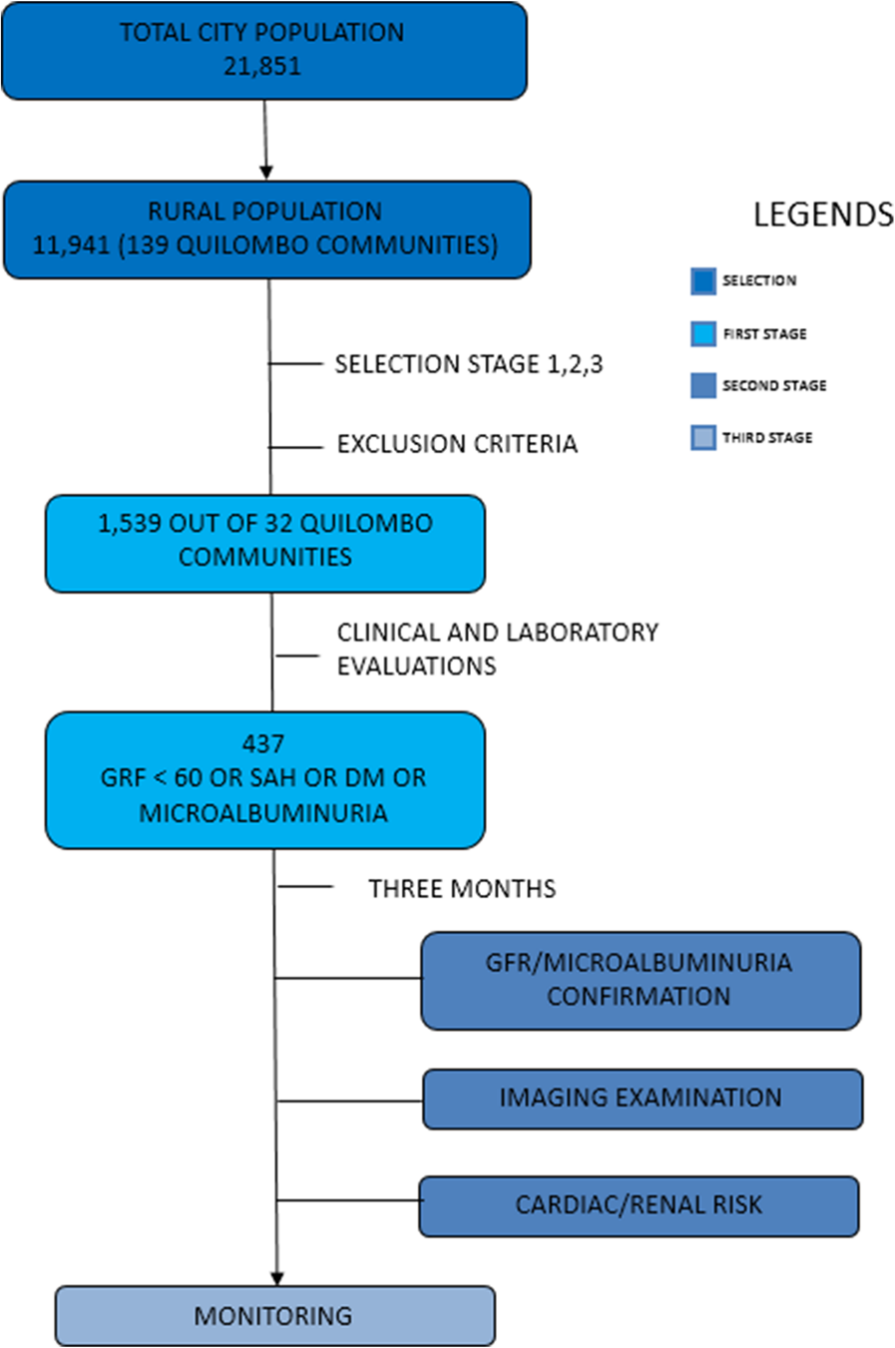
Flowchart of PREVRENAL study stages

This was a cohort study with the following characteristics: population based, prospective, longitudinal, and performed in three stages (Fig. 1). In the first stage, clinical, nutritional, and anthropometric evaluations, measurements of serum and urinary markers for CKD, and examinations for comorbidities (systemic arterial hypertension [SAH], DM, CVD, obesity, and dyslipidemia) were performed. The second stage consisted of repeated examination of individuals with CKD, SAH, and/or DM, image screening, and cardiac risk assessment. The third stage included long-term monitoring of all selected individuals.

### Research team and study timeline

The PREVRENAL team is coordinated by two nephrologist researchers and is composed of: two nephrologists, three cardiologists, two imaging experts (one radiologist and one vascular surgeon), two epidemiologists, four nurses, five nutritionists, two social workers, four biochemists, nine graduation students, and three drivers equipped with four-wheel-drive vehicles. Trips for the first stage’s active search were performed on non-holidayweekends, during a nine-month period from August 2012 to April 2013. Image experts did not participate during this stage. All researchers were trained on the project’s basic procedures.

### First stage

#### Data collection

A questionnaire was completed for each adult in each household. Individual questionnaires were divided into blocks with the following subjects: basic demographic information (age, gender, marital status, and migration); socioeconomic characteristics (education, occupation, working relationship, and income); personal habits (smoking, alcoholism, diet type, and physical exercises); clinical data; arterial blood pressure(AP) status; previous disease of the cardiovascular, renal, and respiratory systems; medications; and health services usage. A minor diet survey was conducted in each family (including sodium content) by nutritionists.

#### Anthropometric measurements and arterial blood pressure

Weight was measured using an electronic scale with 150 kg maximum capacity, and height was measured using a portable anthropometer with 0.1 cm accuracy. Measurements were performed by two independent researchers. Individuals were barefooted, wearing light clothes, and in the orthostatic position. Body mass index(BMI) was calculated and classified according to the International Obesity Task Force (IOTF) criteria. Waist circumference and reference values for gender, age, and ethnicity were defined per NHANES III. AP measurement was performed on the right arm of seated individuals using a proper cuff, which covered 80% of the distance between the olecranon and acromion. The pneumatic bag covered at least 40% of the arm circumference at the level of the heart. Three measurements were performed with a three-minute interval between them and after a five-minute rest; then, the average of the last two measurements was used. The oscillometric method was used for AP assessment using a certified device.

#### Blood and urine collection

Blood was collected after a 12-h fast and the following tests were performed: serum creatinine, urea, cystatin C, glucose, calcium, phosphorus, albumin, cholesterol (total, HDL, and LDL), triglycerides, serum iron, transferrin, glutamic-pyruvic transaminase, ultrasensitive C-reactive protein, and hemoglobin. Total blood and serum samples were collected for freezer storage at − 80 °C. A genetic/ethnic study and evaluations of inflammatory markers (IL-6, TNFα) and APOL 1 polymorphism will be performed using these samples.

Urine sample was collected by the individuals at home and used for testing creatinine, albumin, abnormal elements, and sediment. A second urine collection was used for testing sodium excretion. Estimates for creatinine excretion within 24 h (PrCr24h) and causal urine sodium/creatinine ratio (NaUr) were obtained for estimating the 24-h sodium excretion (Na24h) of isolated samples, per Kawasaki’s Equation [14] (Kawasaki, 1993), which was certified for the Brazilian population.

#### CKD diagnosis

CKD diagnosis was considered for those individuals presenting with a GFR ≤60 mL/min/1.73m^2^ and/or albuminuria > 30 mg/day. The first parameter relates to functional damageand the second indicates structural lesions of the filtration barrier [15, 16] (KDIGO, 2013; LEVEY; BECKER; INKER, 2015). For CKD diagnosis confirmation, GFR and albuminuria tests were repeated three months after the first test. GFR was estimated using four equations, of which three are from the Chronic Kidney Disease - Epidemiologic Collaboration Equation (CKD-EPI) study as follows: 1) one equation based on creatinine values [17]; 2) one equation based on cystatin C values; and 3) one equation based on both creatinine and cystatin values [18]. Serum creatinine was measured using the modified Jaffé’s method. Immunoturbidimetry was used to measurecystatin C.

### Second stage

#### Cardiovascular risk

Cardiovascular risk assessment was performed using Framingham’s risk score [19]. Arteriosclerosis was also evaluated using two-dimensional ultrasonography of the carotid arteries and computerized tomography of the coronary arteries, in addition to tomographic measurement of visceral fat.

##### Carotid artery two-dimensional ultrasonography

Carotid intima-media thickness (CAIMT) was measured using an ultrasonography device (Vivid3, Vingmed GE, Horten, Norway). Examination was performed by a single experienced blinded examiner, who was not aware of the patient’s clinical and laboratory information, and risk classification. Bilateral carotid system evaluation was performed after at least 10 min of rest in the supine position with the neck in slight hyperextension. CAIMTwas measured at the artery wall of the common carotid, most distal to the transducer and 1 cm from the artery bifurcation. Measurements consisted of the distance between two echogenic lines traced at the lumen-intima and media-adventitia interfaces, and were considered normal when CAIMT< 0.9 mm, and abnormal when CAIMT > 0.9 mm [20].

##### Abdominal computerized tomography (CT)

Multi-slice helical CT was performed using a 64-channel CT scanner (Aquilion, Toshiba, Tokyo, Japan). First, a 10 mm unique volumetric section is axially performed under apnea (equivalent to 34 × 0.29-mm thick submillimeter cuts). A tomographic section of the lumbar region at the navel level, corresponding to the L3-L4 height, is usually topographically equivalent to the renal hilum projection (variation is defined depending on each patient’s renal hilum height). Examination was performed with a 0.4–0.6 s exposure time, field of view corresponding to the sample’s frame size (between 320 and 440 m), and 120 kV energy potential at 340 mAs.

Visceral fat analysis was performed at the workstation, using appropriate and specific software for the CT scanner, capable of differentiating all abdominal structures contained in the obtained volumetric section. Subsequently, each section’s tridimensional reconstruction was obtained, where all structures and organs are displayed through a color scale that is subjectively standardized by the researcher, facilitating tissue visualization, e.g. visceral fat. After the reading and design stages, quantitative abdominal visceral fat calculation is automatically performed by the software and expressed as volume per mL. Section points were defined as ≤102.5 cm^3^ (mL) for men and ≤84.1 cm^3^ (mL) for women [21].

##### Computerized tomographyof coronary arteries

Images were obtained using the same equipment. Each patient was placed in the supine position, and 3.0-mm thick (on average) images were obtained without intervals. Calcium presence was measured when the density was above 130 Hounsfield units in at least three continuous pixels (>1mm^2^) of the same artery. The coronary calcium score was composed of the sum of individual scores from the left and right coronary arteries and was analyzed and divided into categories according to the methods ofMcCellandet al [22].

##### Cardiac and renal echo-Doppler

All individuals with reduced GFR, SAH, DM, and/or high albuminuria were referred for echo-Doppler evaluation (Vivid3, Vingmed GE, Horten, Norway), with second harmonic and sectorial electronic transducer at 2–4 MHz. Cardiac and renal evaluations were performed. Cardiac evaluation consisted of thoracic echo-Doppler cardiogram, in which the following parameters were analyzed: ventricular geometry, systolic function, and diastolic function, according to the American Society of Echocardiography (ASE) recommendations [23, 24]. Renal evaluations consisted of obtaining the intrarenal resistive index, using renal Doppler, according to the methods ofRadermacher [25] (2002). In summary, analysis was performed using pulsed wave Doppler, obtaining the peak systolic (Vmax) and diastolic (Vmin) velocities; the index was calculated using the following equation: 100 x [1 - (Vmin/Vmax)]. The intrarenal resistive index (IRI) was calculated as the average of measurements performed for the upper, medium, and lower segments of both kidneys.

#### Chronic kidney disease (CKD) confirmation

All individuals with reduced GFR, SAH, DM, and/or high albuminuria were re-examined after three months in order to confirm the diagnosis of CKD.

### Third stage

All individuals selected for the first stage will be re-visited and all examinations will be repeated five years after the first stage.

### Statistic analysis

First, a descriptive analysis of the variables under study will be performed. Quantitative variables will be presented by mean and standard deviation and qualitative variables by frequencies and percentages. The Shapiro Wilk test will be used to evaluate the normality of the quantitative variables.

For categorical variables, group comparison will be based upon the results of the Chi-square test. Chi-square test will be replaced by Fisher’s exact test if the expected frequency in any of the cells of the contingency table is less than 5. For continuous variables, group comparison will be based upon the results of the *t*-test or the Wilcoxon rank-sum test, depending on the normality.

In the primary analysis, the prevalence of CKD at baseline and its corresponding 95% confidence interval (CI) will be presented. Prevalence of hypertension, diabetes mellitus, cardiovascular disease and other comorbidities at baseline and their corresponding 95% CIs will also be presented.

In the secondary analysis, the factors associated with the presence of CKD through bivariate analysis with an unadjusted prevalence ratio estimate and a 95% confidence interval (95% CI) will be used. The independent variables that present a significance lower than 0.20 (*p*-value < 0.20) will be considered in the final model, that is, in the adjustment of potentially confounding variables through the multivariate logistic regression technique performed step by step.

The comparison of survival (based on all-cause mortality) between patients with and without CKD will also be performed based upon the log-rank test performed at a significance level of 0.05. Hazard ratios and 95% CIs will be calculated by a Cox proportional hazard regression model.

The data will be analyzed in the statistical program STATA 12.0.

### Ethical considerations

This study is being conducted in accordance with the principles established by the 18th World Medical Assembly (Helsinki, 1964) and all subsequent amendments.

The study was approved by the Research Ethics Committee of the University Hospital of the Federal University of Maranhão (n° 41,492/2012). Written informed consent for participation in the study was obtained from all participants.

## Discussion

Brazil lacks early-stage CKD population study data. The prevalence of CKD in our country is unknown for both the overall and African descent populations. After identifying individuals with reduced GFR and high albuminuria, in an isolated population of African descendants, this study proposes to monitor patients with or without these conditions during long-term follow-up. This study also seeks to identify other associated clinical conditions, including SAH, DM, anemia, dyslipidemia, metabolic syndrome, and cardiovascular risk. There are no specific recommendations for detecting CKD in African descendants [26, 27], thus four equations for estimating GFR based on serum creatinine and cystatin were used and will be compared.

To date, there are no reports on arteriosclerotic disease and its relationship with GFR among Brazilian African descendants. Data concerning clinical and laboratory factors related to the development of arteriosclerosis among individuals with impaired renal function during pre-uremia stages and understanding of thisprocess’ development will allow the adoption of therapeutic and preventive measures that enable reduction of cerebrovascular and coronary events, which result in high morbidity and mortality among this group of patients. Additionally, our study is the first to quantify and evaluate factors associated with coronary calcification in African descendants who descend from African slaves and live in isolated Brazilian communities, filling a gap of this research area.

Differences in CVD occurrence among ethnic groups cannot be completely explained by traditional risk factors, even when taking into account the increased rate ofSAH and DM among African descendants [28]. Thus, the association between arteriosclerosis and CKD among the various ethnic groups and impacts of risk factors on this association are not well-established. Therefore, the American Heart Association has recommended investigations of arteriosclerotic CVD among all ethnic groups, including long- and short-term risk analyses, in order to fill this knowledge gap [29].

In addition to common ancestry, other factors contribute to the homogeneity of the studied population, e.g. geographic and social isolation. Food habits of the studied population are also similar and reveal the habit of conserving majority of perishable foods using salt, especially meat, fish, and other seafood. Food sodium content was evaluated through a food survey and quantified through urinary sodium excretion tests. Local populations secure subsistence by cultivating manioc (*Manihotesculenta*) for flour production, hunting, fishing, and native fruits extractivism, e.g. babaçu (*Orbignyaphalerata*), juçara (*Euterpeoleracea*), and buriti (*Mauritiaflexuosa* L.). Socioeconomic status is equally low, a remarkable characteristic of the majority of the population of the State of Maranhão, which represents the lowest human development index level and the lowest life expectancy in comparison with other Brazilian states.

This study has some limitations. 1) It will be impossible to properly monitor (every five years) the entire sample (1539 individuals) due to difficulty of access to quilombo communities. 2) A highly specific group will be monitored; thus, results cannot be automatically extrapolated to the entire Brazilian African decent population. 3) Patients’ outcome will not follow a natural course, since therapeutic interventions will occur as risk factors are identified or diseases are diagnosed. 4) Finally, it is impossible to reach the gold standard for urinary sodium excretion quantification, due to difficulties in collecting urine every 24 h.

In contrast, our study has the main positive aspect of being the first study to quantify and evaluate factors associated with kidney diseases in African populations who descend from African slaves and live in isolated Brazilian communities, providing missing information to this area of research.

The PREVRENAL study describes characteristics of the target population, selection of individuals, and detection of a population at risk, according to applied clinical and laboratory evaluations. The methodology of imaging examinations applied to the population at risk is also described. The first and second stages were concluded and results will be published in specific journals appropriate for each knowledge area. The subsequent stage is the long-term and continuous monitoring of individuals diagnosed with renal abnormalities, as well as those with CKD risk factors, especially SAH and DM. The entire sample will be re-evaluated five years after study initiation. The expectation is to obtain information about CKD evolution among this ethnic group, including progression rate, development of complications (e.g. anemia, mineral and bone disorder, acidosis, and uremia symptoms), and occurrence of cardiovascular events, which has a high impact on morbidity and mortality of CKD patients.

Therefore, we expect to contribute new information about CKD development and progression within a specific population group that represents the base of the Brazilian population. Results and reports of each topical area will be sent to government branches responsible for managing the national and local Unique Health System (SUS - SistemaÚnico de Saúde), in order to assist the establishment of public policies.

## Abbreviations

AP: arterial blood pressure
CAIMT: carotid intima-media thickness
CKD: chronic kidney disease
CKD-EPI: Chronic Kidney Disease - Epidemiologic Collaboration Equation
CVD: cardiovascular disease
DM: diabetes mellitus
GFR: glomerular filtration rate
PREVRENAL: Prevalence of chronic kidney disease and comorbidities in isolated African descent communities
SAH: systemic arterial hypertension
